# Household economic burden of managing people living with HIV and comorbidities of hypertension and diabetes in La Nkwantanang-Madina Municipality, Ghana

**DOI:** 10.1371/journal.pgph.0004371

**Published:** 2025-04-07

**Authors:** Richmond Owusu, Esther Esi Degbor, Desmond Dzidzornu Otoo, Ruby A. M. Annan

**Affiliations:** Department of Health Policy, Planning and Management, School of Public Health, University of Ghana, Accra, Ghana; University of Colorado Anschutz Medical Campus: University of Colorado - Anschutz Medical Campus, UNITED STATES OF AMERICA

## Abstract

Managing HIV alongside chronic conditions such as hypertension and diabetes present significant economic challenges for households, especially in low-and middle-income countries. These chronic diseases not only reduce the quality of life of people living with HIV (PLHIV) but also further increase their economic burden. This study seeks to examine the economic burden of managing PLHIV with hypertension and diabetes comorbidities. The cross-sectional cost-of-illness study used quantitative data gathered from 56 PLHIV with hypertension and/or diabetes receiving ART at two Polyclinics and the Pentecost Hospital in the La Nkwantanang-Madina Municipality. A structured questionnaire was used to collect data on patients’ socio-demographic characteristics, direct, indirect, and intangible costs between September to December 2023. Data was analyzed and presented descriptively.The total economic cost of PLHIV managing comorbidities was GHS11,892.11 (USD 1,022.54) with a direct cost of GHS10,739.00 (US$ 923.39) accounting for 90.3% and indirect cost of GHS 1,153.14 (US$ 99.15) accounting for 9.7% over 6 months. Direct medical costs constituted 83.1% of total costs with the cost of medicines being the largest cost component. There was a significant difference between the total costs across the comorbidities (X^2^=8.58, p = 0.0137). Approximately 45.24% of the average annual income per person was spent on managing comorbidities in PLHIV. About 89% of participants reported a low intangible cost burden. This study reveals the significant economic burden on households managing HIV with hypertension and diabetes comorbidities. Direct costs driven by medication expenses constituted the majority of the burden, while productivity losses compounded indirect costs. Despite widespread health insurance coverage, substantial out-of-pocket payments are made in the management of these comorbidities. The findings emphasize the need for integrated healthcare strategies to address both communicable and non-communicable diseases, especially in low-income settings, and policies to reduce financial barriers. Studies should explore long-term burden and strategies to alleviate the economic impact on vulnerable populations.

## Introduction

The burden of Human Immunodeficiency Virus (HIV), hypertension, and diabetes presents a significant challenge to healthcare systems especially in low-and middle-income countries (LMICs). The emergence of HIV has drawn significant global attention, especially with its great human impact and consequential ramifications on economies and societies [1].

According to the joint United Nations programme on HIV/AIDS (UNAIDS), about 39.9 million people were living with HIV with 1.3million new infection in 2023 with most cases living in low- and middle-income countries [2]. As of 2022, an estimated 42.3 million people had died from Acquired Immune Deficiency Syndrome (AIDS) related causes [3]. Research findings indicate that sub-Saharan Africa is the most significantly impacted region [4] with Africa having about 67% to 70% of the disease burden [4,5]. In Ghana, despite a decrease in the prevalence of HIV from 2.4% in 2016 to 1.5% in 2023, there were a total of 330, 000 people living with HIV (PLHIV) as of 2023 [6,7].

Advancements in antiretroviral therapy (ART) has reduced the fatality of HIV infection from an acute infection to a manageable chronic disease [8]. Data from UNAIDS [2] suggests that about 30.7 million infected people are now accessing ART globally compared to 7.7 million in 2010. PLHIV are living longer [9], however, as they age, they are often at greater risk of poorer health outcomes compared to the general population due to their higher susceptibility to the development of additional chronic comorbidities, such as type 2 diabetes, hypertension, tuberculosis, depression, and some cancers [10]. It is reported that these comorbidities are common among the aging population and particularly among those living with HIV [11,12]. PLHIV experience a higher prevalence of non-HIV related comorbidities, such as alcohol abuse, chronic renal disease, cardiovascular disease, dyslipidemia, and hepatitis B and C, compared to non-HIV controls [10].

Managing these chronic conditions simultaneously places a substantial economic burden on households, exacerbating the financial strain already posed by HIV management.

Hypertension, which is a medical condition characterized by continually elevated arterial blood pressure is a common comorbidity among PLHIV, with a reported global prevalence of 23.6% [13]. Diabetes, which is a persistent and metabolic ailment distinguished by heightened levels of blood sugar, affects about 529 million people worldwide with the majority residing in LMICs [14]. In Ghana, the diabetic population is estimated to encompass approximately 2.4 million individuals, while Type-2 Diabetes is prevalent among approximately 7.5% of the adult population [15]. The disease burden of diabetes among HIV patients is a significant concern with the co-occurrence of type 2 diabetes and HIV increasing the economic burden at both the patient and country level [16].

The National Health Insurance Scheme (NHIS) plays a crucial role in reducing out-of-pocket healthcare expenditure to improve access to essential services. While the NHIS has improved access to care, it often does not cover all expenses associated with the management of other comorbidities such as hypertension and diabetes. The prevalence of HIV-associated comorbidities has been shown to drive the cost of health care [10], causing an economic burden on households and the economy at large. The economic burden of disease focuses on the financial costs of illnesses for individuals, caregivers, households, and societies [17]. In Ghana, research has revealed that HIV/AIDS significantly hinders economic growth over the short and long term [18] and households who have HIV/AIDS diagnosed member(s) incur higher expenditure for health services [19]. It is reported that, for each 1% increase in HIV/AIDS prevalence, there is a 0.47% decrease in per capita income in sub-Saharan Africa [20]. In Ghana, some studies [21,22] have examined the economic burden of HIV on households. These studies, however, did not consider the cost of comorbidities associated with HIV.

With the rising prevalence of NCDs, especially among PLHIV, understanding the household economic burden of managing both HIV and its comorbidities is crucial for informing health policies and interventions aimed at reducing financial hardship for affected families. This study seeks to quantify the household economic burden of managing PLHIV with comorbid hypertension and diabetes in La Nkwantanang-Madina Municipality.

## Materials and methods

### Ethics statement

Ethical approval for the research was obtained from Ghana Health Service Ethics Review Committee with number GHS-ERC:066/07/23. Permission was sought from the management of the three study sites. Written informed consents were obtained from all participants before data collection.

### Study design

The study employed a cross-sectional cost-of-illness analysis technique in estimating the economic burden of HIV and comorbidities of hypertension and diabetes among people living with HIV [23,24]. A quantitative method was used to examine the direct, indirect, and intangible costs of HIV and comorbidities of hypertension and diabetes from the patients’ perspective.

### Study area

This study was conducted in three health facilities within the La Nkwantanang-Madina Municipality. The municipality is one of the 29 districts in the Greater Accra Region. The municipality has a population of 244,676 people, according to the 2021 Population and Housing Census [25]. The Ghana AIDS Commission estimated the HIV prevalence among the adult population (18+ years) in the municipality as 6.60% in 2019 [26]. The selected facilities included two polyclinics Madina Polyclinic, Kekele, and Madina Polyclinic, Rawlings Circle and the Pentecost Hospital. These facilities are strategically located in densely populated areas, have well-functional ART units that provide specialized care and established diabetic and hypertension clinics with laboratory diagnostic capabilities to manage these conditions.

### Study population

The target population were adults (18+ years) living with HIV, diagnosed with either hypertension, diabetes, or both and actively receiving care at the ART clinics of the Madina Polyclinics (Kekele and Rawlings Circle) and the Pentecost Hospital.

### Inclusion criteria

Participants included in the study were adult (18 years and above) patients attending ART clinic, diagnosed of HIV and either hypertension, diabetes or both irrespective of sex, culture, background, religious belief and who gave their consent to participate in the study. The patient must have been on ART treatment for at least six (6) months at the time of data collection.

### Exclusion criteria

The study excluded patients with HIV, diabetes, and/or hypertension comorbidities who were too ill or hospitalized during the period of data collection. Patients who declined to participate were excluded.

### Study variables

The primary outcome variable in the study was the total economic burden associated with managing HIV and comorbidities of hypertension and diabetes. The explanatory variables were direct, indirect, and intangible costs. Table 1 represents a description of variables of interest and their components. Socio-demographic variables such as sex, age, marital status, educational level, religion, and average monthly income were also collected.

**Table 1:** Study Variables.

Type of Variable	Cost type	Cost Category	Description
Primary Outcome	Economic Burden	Total cost Average cost	Sum of all costs for managing HIV and comorbidities of hypertension and diabetes
Explanatory outcomes	Direct cost	Medical cost	Consultation Medication Laboratory tests
		Non-medical cost	Transportation Food and drink Others
	Indirect cost	Productivity losses	Productivity losses (working hours lost) Waiting time Travelling time
	Intangible cost	Intangible burden	Physical pain Social isolation Anxiety Stress

### Sampling

Purposive sampling technique was employed to select participants who had HIV with hypertension and/or diabetes comorbidities for the study. This technique was used because of the narrow nature of the population of interest, as it allows researchers to focus on cases that can provide the most pertinent data. Participants were recruited from 11^th^ September to 30^th^ November 2023 from the three (3) health facilities’ ART clinics. A total of Fifty-six (56) PLHIV with hypertension and/or diabetes comorbidities were finally recruited in the study as follows: 19 from Madina Polyclinic, Kekele; 15 from Madina Polyclinic, Rawlings Circle; and 22 from Pentecost Hospital.

### Data collection tools and procedure

Structured questionnaires were used to collect data from respondents using a face-to-face interview technique. The questionnaire was originally designed and administered in English language and translated to local dialects (Ga, Akan, and Ewe) for some participants. The questionnaire covered sociodemographic characteristics, health status and treatment information, direct cost information, indirect cost information and intangible cost information. Retrospective cost data was collected for the past 6 months.

Participants were selected during their visits to the ART clinics at the three selected healthcare facilities from 11^th^ September to 30^th^ November 2023. All participants were given detailed information about the study. Interviews were conducted in a private and quiet area within the ART clinics to ensure privacy, confidentiality and minimize distractions. Each interview lasted for about 30minutes and was conducted by the researcher and assistants. The data collected were double-checked for completeness and accuracy before entry into Microsoft Excel 2016. It was cleaned to correct errors, coded, and exported into STATA version 17.

### Data quality assurance

To ensure data quality, two (2) research assistants who received a 2-day training on research methods, questionnaire administration as well as ethical guidelines were involved in the data collection process. The questionnaire was pre-tested at the LEKMA Polyclinic, which has a similar working environment and HIV counselling and ART clinic as the Madina Polyclinics and Pentecost Hospital. Feedback from the pre-test was used to refine the questionnaire, to capture all necessary data and to make it user-friendly. Recall bias was minimized through the limitation of the recall period to 6 months and the use of structured questionnaires to guide participants. Also, participants were encouraged to refer to available receipts to verify expenses and in cases where receipts were not available, most recent clinic expenses were used for estimates.

### Data analysis

Data entered into Microsoft Excel 2016 was exported into STATA version 17 for analysis. Data was analyzed descriptively and presented as frequencies, percentages, bar and pie charts. A Kruskal-Wallis test was conducted to compare difference in cost across the comorbidities. Percentage of GDP per capita was calculated using Ghana’s 2023 GDP per capital of US$ 2260.3.

### Estimation of direct Cost

This was estimated by summing all direct medical and non-medical costs of managing HIV and comorbidities. The direct medical cost was estimated by summing all cost of consultation, medicines/drugs, laboratory and other expenses incurred by participants over the last six months. Direct non-medical costs were estimated by summing all costs on transportation to and from the health facility, cost of food and drink and other related costs due to the disease conditions. Transportation cost covered the cost of caregivers and patients for regular hospital review and treatment. The total direct cost was obtained by summation of all components of medical and non-medical expenses made by the participant due to HIV and the comorbidities.

### Estimation of indirect costs

The human capital method was adopted to estimate the indirect cost. The human capital approach assigns monetary value to time lost from productive activities by patients and their caregivers, with the assumption that time loss represents lost economic output [27,28]. This method was used to place monetary value on the lost productivity due to illness from HIV, hypertension, and diabetes and the time spent by caregivers. The total indirect cost was estimated by summing the waiting time at the hospital, travel time to and from the hospital and number of days absent from productive work by the patients over the last six (6) months as well as their caregivers multiplied by the national minimum wage. Productivity loss was therefore valued using the 2023 national minimum wage (GHS14.88) in Ghana [29]. This was to provide a standardized measurement to productivity loss or a baseline value that can be applied to all participants since actual earnings could be inconsistent or underreported thereby avoiding bias due to income variability. Thus, the average daily income was used in the sensitivity analysis. Productivity hours lost for both patients and caregivers were calculated by multiplying the number of workdays lost due to absenteeism, travel, and waiting time by 8hours (standard working hours per day in Ghana).

### Estimation of intangible costs

Intangible cost was estimated using a five-dimension Likert scale where study participants were asked to score statements under each dimension, i.e., 1= None, 2=Very little, 3=Moderately, 4=Severe, 5=Extremely severe, in respect of physical pain, anxiety, stress, and social isolation. Composite scores were obtained by summing up the dimensions in each domain and multiplying each by the number of questions. Using the descriptive tertile approach, the composite scores were then reclassified into Low (7-13), Moderate (14-19), and High (20 and above) intangible costs.

### Estimation of total economic burden

The total economic burden of PLHIV and comorbidities of hypertension and diabetes was estimated by summing the total direct cost and total indirect cost incurred by participants over the 6-month period. The total direct cost included all medical expenses and non-medical expenses. Indirect costs were made up of a valuation of productivity loss, in instances where recall was difficult, the total treatment cost for the last visit to the clinic was considered to be the monthly cost during the past six months as the participants visited the facility on schedule (i.e., monthly, quarterly or half annually).Therefore, in estimating the annual household economic cost of managing HIV with comorbidities of hypertension and diabetes, the estimated total economic costs for the six months was multiplied by 2 assuming that participants maintained consistent utilization patterns throughout the year. The average cost per participant was estimated by dividing the total economic costs by the number of study participants. Also, the relative contributions (cost distribution) of direct and indirect costs to the total economic burden were calculated and expressed as percentages.

### Sensitivity analysis

One way sensitivity analysis was conducted on two components: the cost of medications and daily income of study participants. These variables were chosen due to the inherent uncertainty surrounding their estimates. The cost of medications was varied by 25% to account for potential fluctuations in drug prices, while daily income was similarly adjusted to assess the robustness of the findings to changes in participants’ income levels. A variability of 25% was adopted as an average range of variability observed in medication pricing under the NHIS. In 2023, the prices of medicines increased between 20% to 30% under the NHIS [30].

## Results

### Socio-demographic characteristics of study participants

Table 2 displays the socio-demographic characteristics of the study participants. The mean age of the study participants was 53.20 ± 11.1 years with the majority 44.6% (n = 25) aged 50 to 59 years. Approximately three out of every four of the study participants were females (76.8%). Most participants had at least basic education level, with 28.6% (n=16) completing Junior High School (JHS) and 25.0% (n=14) attaining Senior High School, vocational, or technical school education. As much as 21.4% (n=12), had no formal education. Furthermore, 44.6% (n=25) were married. In terms of employment, more than half of the respondents 64.3% (n = 36) were employed with only 14.3% (n = 8) being in the formal sector while 50% (n = 28) were in the informal sector with a mean income of GHS 979.96 (95% CI: 778.29 – 1,181.64). Half of the respondents 50% (n = 28) earned a monthly income between GHS500 - GHS1,000. About 94.6% (n = 53) of the participants were insured under the National Health Insurance Scheme (NHIS).

**Table 2:** Socio-demographic characteristics of study participants.

Variable	Frequency	Percentage (%)
**Age group (years):**		
20 – 29	1	1.8
30 – 39	2	3.6
40 – 49	13	23.2
50 – 59	25	44.6
60 and above	15	26.8
**Mean age: 53.20 ± 11.1**		
**Sex:**		
Male	13	23.2
Female	43	76.8
**Level of Education:**		
None	12	21.4
Primary	10	17.9
JHS	16	28.6
Senior High/Vocation/Technical School	14	25.0
Tertiary	4	7.1
**Marital status:**		
Never married	1	1.8
Married	25	44.6
Co-habiting	2	3.6
Widowed	16	28.6
Divorced/Separated	12	21.4
**Employment status:**		
Employed	36	64.3
Unemployed	8	14.3
Retired/Pension	11	19.6
Student/Apprentice	1	1.8
**Employment sector:**		
Formal	8	14.3
Informal	28	50.0
Unemployed	20	35.7
**Monthly income (GHS):**		
< 500	12	21.4
500 – 1000	28	50.0
> 1000	16	28.6
**Mean monthly income (GHS) 979.96 (95% CI: 778.2905 - 1181.638)**		
**Health insurance status:**		
NHIS	53	94.6
Uninsured	3	5.4

### Health status and treatment information of participants

As shown in Table 3, the median number of visits was 5, with a minimum of 1 and a maximum of 12 visits in the past 6 months. About 57% (n=32) of the participants had hypertension only as a comorbidity while those with hypertension and diabetes comorbidities constituted about 14.3% (n = 8). Of the respondents with hypertension, the majority (65.6%) had lived with the condition for less than 5 years while 6.3% (n=2) had lived with the condition for between 10-20 years and the mean years respondents had lived with HIV and hypertension was 4.38 years (CI: 3.06 - 5.70). About half of the respondents (50.0%) with diabetes as a comorbidity had lived with the condition for less than five years. Out of the participants living with hypertension and diabetes comorbidities, about 62.5% (n=5) had lived with the conditions for between 10-20 years. Additionally, out of the total number of participants who were non-adherent to medications, 12.5% (n=7) had hypertension only, while only 1.8% (n=1) had diabetes only.

**Table 3:** Health status and treatment information of participants.

Variable	Frequency	Percentage (%)
**Number of visits:** Median = 5, Min= 1, Max = 12		
**Average number of visits:** Hypertension: 5.15, Diabetes: 5.10, Both: 5.31		
**Distribution of people living with comorbidity:**		
Hypertension only	32	57.1
Diabetes only:	16	28.6
Both hypertension and diabetes	8	14.3
**Period living with comorbidity (Years):**		
Hypertension only:		
< 5	21	65.6
5 – 9	9	28.1
10 – 20	2	6.3
**Mean (Years) 4.38 (CI: 3.06 - 5.70)**		
Diabetes only:		
< 5	8	50.0
5 – 9	8	50.0
**Mean (Years) 3.94 (CI: 2.83 - 5.05)**		
Both hypertension and diabetes:		
< 5	1	12.5
5 – 9	2	25.0
10 – 20	5	62.5
**Mean (Years) 11.94 (CI: 7.94 - 15.94)**		
**Treatment adherence (comorbidities):**		
**Hypertension only:**		
Adherence	25	44.6
Non-adherence	7	12.5
**Diabetes only:**		
Adherence	15	26.8
Non-adherence	1	1.8
**Both hypertension and diabetes:**		
Adherence	8	14.3
Non-adherence	–	–
**Total**	**56**	**100.0**

### Direct cost of managing PLHIV and hypertension and diabetes

Table 4 shows the distribution of direct costs for the last six months’ hospital visitation by respondents’ status. The direct medical cost accounted for about 83.1% of the total cost of GHS8,919.00 (US$766.90). Medicine cost was a major cost driver for the total direct medical cost of health care for those with hypertension (32.3%), diabetes (15.8%), and both hypertension and diabetes (24.7%). PLHIV and hypertension spent an estimated GHS3,474.00 (US$ 298.71) on medicines, HIV and diabetes spent GHS1,700.00 (US$146.17) while HIV with hypertension and diabetes spent GHS2,650.00 (US$227.86). The mean medicine cost for hypertension only, diabetes only and both hypertension and diabetes were GHS108.56 (US$9.33) (95% CI: 69.63 - 147.49), GHS106.25 (US$9.14) (95% CI:78.98 - 133.52), and GHS331.25 (US$28.48) (95% CI: 56.64 - 605.87) respectively with median GHS 80 (IQR 50-100), GHS 87.5 (IQR: 70-150) and GHS 155 (IQR: 150-470) respectively. Direct non-medical costs during the last six months accounted for about 16.9% of the total direct cost at an estimated cost of GHS1,820.00 (US$156.49) with a mean cost of GHS32.50 (US$2.79) (95% CI: 25.95 - 39.01) and median cost of GHS29 (IQR: 12-40). Patients with HIV and hypertension recorded the highest total travel cost, GHS879.00 (US$75.58) while the lowest total travel cost GHS266.00 (US$22.87) was accounted for by HIV with both hypertension and diabetes participants. However, the highest median travel cost was observed for patients managing both hypertension and diabetes (GHS 35, IQR: 23-40), compared to hypertension only (GHS25, IQR: 10-32.5) and diabetes only (GHS 16, IQR: 10.5-40).

**Table 4:** Direct medical cost of Managing PLHIV and Hypertension and Diabetes.

Cost item	Cost		Mean (GHS)	95% CI (GHS)	Median (IQR) GHS	Cost profile (%)
	GHS	USD				
**Direct Medical Cost**						
**Hypertension only:**						
Consultation	50.00	4.30	1.56	-1.62 - 4.75	0(0-0)	0.5
Lab Tests	405.00	34.82	12.66	1.16 - 24.15	0(0-0)	3.8
Medicines	3474.00	298.71	108.56	69.63 - 147.49	80(50-110	32.3
**Subtotal**	**3929.00**	**337.83**	**122.78**	**82.84 - 162.73**	**87.5 (50-150)**	**36.6**
**Diabetes only:**						
Consultation	–	–	–	–		–
Lab Tests	330.00	28.37	20.63	4.18 - 37.07	0(0-45)	3.1
Medicines	1700.00	146.17	106.25	78.98 - 133.52	87.5(70-150)	15.83
**Subtotal**	**2030.00**	**174.55**	**126.88**	**86.89 - 166.86**	**107.5 (70-150)**	**18.9**
**Both hypertension and diabetes:**						
Consultation	–	–	–	–		–
Lab Tests	310.00	26.66	38.75	4.01 - 73.49	35(0-80)	2.9
Medicines	2650.00	227.86	331.25	56.64 - 605.87	155(150-470)	24.7
**Subtotal**	**2960.00**	**254.51**	**370.00**	**113.95 - 626.05**	**235 (225-470)**	**27.6**
**Total direct medical cost**	**8919.00**	**766.90**	**159.27**	**114.51 - 204.03**	**102.5 (70-225)**	**83.1**
**Direct non-medical cost**						
**Hypertension only:**						
Travel cost	879.00	75.58	27.47	20.41 - 34.53	25(10-32.5)	8.2
Food and drinks	208.00	17.88	6.50	2.81 - 10.19	2.5(0-7.5)	1.9
Others	1.00	0.09	0.03	-0.03 - 0.095	0(0-0)	0.0
**Subtotal**	**1088.00**	**93.55**	**34.00**	**24.75 - 43.25**	**27.5 (12.5-52.5)**	**10.1**
**Diabetes only:**						
Travel cost	350.00	30.09	21.88	13.71 - 30.01	16(10.5-40)	3.3
Food and drinks	40.00	3.44	2.50	-5.77 - 5.58	0(0-0)	0.4
Others	1.00	0.09	0.01	-0.71 - 0.20	0(0-0)	0.0
**Subtotal**	**391.00**	**33.62**	**24.39**	**14.49 - 34.39**	**16 (10.5-40)**	**3.6**
**Both hypertension and diabetes:**						
Travel cost	266.00	22.87	33.25	16.47 - 50.04	35(23-40)	2.5
Food and drinks	74.00	6.36	9.25	-2.85 - 21.35	1.5(0-15.5)	0.7
Others	1.00	0.09	0.13	-0.17 - 0.42	0(0-0)	0.0
**Subtotal**	**341.00**	**29.32**	**42.63**	**19.58 - 65.67**	**40 (30-55)**	**3.2**
**Total direct non-medical cost**	**1820.00**	**156.49**	**32.50**	**25.95 - 39.01**	**29 (12-40)**	**16.9**
**Total**	**10739.00**	**923.39**	**191.77**	**143.96 - 239.57**	**132.5 (90.5-252)**	**100.0**

USD 1 = GHS 11.63 Bank of Ghana Interbank rate as of December 2023.

### Comparison of total direct costs across comorbidities

A Kruskal-Walli’s test conducted to compare the total direct costs between hypertension only, diabetes only and both hypertension and diabetes comorbidities showed a significant difference in total cost across the three comorbidities (X^2^=8.58, p = 0.0137). (Table 5).

**Table 5:** Comparison of costs across comorbidities.

Comorbidity	Rank sum	Chi^2^	df	p-value
Hypertension only	822.50			
Diabetes only	420.50			
Both hypertension & Diabetes	353.00			
		**8.580**	**2**	**0.0137**

A post-hoc pairwise comparison in Table 6 showed a significant difference in the total costs between patients with both hypertension and diabetes and hypertension only (z= -2.90, p=0.0038) and patients with both conditions and diabetes only (z= -2.42, 0.0155).

**Table 6:** Post-hoc comparisons.

Comparison	z-statistic	p-value
Hypertension vs. Diabetes	-0.09	0.9303
Hypertension vs. Both Hypertension & Diabetes	-2.90	0.0038
Diabetes vs. Both Hypertension & Diabetes	-2.42	0.0155

### Indirect cost of managing PLHIV and hypertension and diabetes

Table 7 shows that out of total 649 productive hours lost, about 33.2% (206 hours) was attributed to patients who have hypertension only as comorbidity while about 9.7% (59 hours) was attributed to patients who have both hypertension and diabetes as comorbidities. The total number of days absent from work by the participants due to the diseases was highest at about 19 days out of 6 months for participants with hypertension only as a comorbidity while the least (2 days) is attributed to patients with diabetes only as a comorbidity. Out of the total productive days lost in the last 6 months, participants lost 37 productive days while caregivers lost 40 productive days while accompanying participants to the clinic for treatment. Participants with hypertension only as comorbidity lost 19 productive days valued at GHS282.72 (US$24.31) to absenteeism. The total productive days lost to participants and accompanying caregivers due to absence from work, waiting time and travel time was estimated at 77 days. The total productive days lost was valued based on the national minimum wage (GHS14.88), hence, the total valued productive days lost was estimated at GHS1,153.14 (USD 99.15).

**Table 7:** Indirect Medical Cost of Managing PLHIV and Hypertension and Diabetes.

Item	Category	Hours lost	Days lost^ª^	Valued productive days lost		Cost profile (%)
				GHS^b^	USD^c^	
Patients:						
Hypertension only	Absenteeism	152	19	282.72	24.31	24.5
	Travel time	47	6	86.96	7.48	7.5
	Waiting time	7	1	12.80	1.10	1.1
**Subtotal**	**–**	**206**	**26**	**382.48**	**32.89**	**33.2**
Diabetes only	Absenteeism	47	2	29.76	2.56	2.6
	Travel time	16	2	29.14	2.51	2.5
	Waiting time	3	0	5.12	0.44	0.4
**Subtotal**	–	**65**	**4**	**64.02**	**5.50**	**5.6**
Both hypertension and diabetes	Absenteeism	47	6	89.28	7.68	7.7
	Travel time	10	1	17.98	1.55	1.6
	Waiting time	2	0	4.19	0.36	0.4
**Subtotal**	**–**	**59**	**7**	**111.45**	**9.58**	**9.7**
Caregivers:						
Hypertension only	Absenteeism	160	20	297.60	25.59	25.8
Diabetes only	Absenteeism	96	12	178.56	15.35	15.5
Both hypertension and diabetes	Absenteeism	64	8	119.04	10.24	10.3
**Subtotal**	–	**320**	**40**	**595.20**	**51.18**	**51.6**
**Total**	–	**649**	**77**	**1,153.14**	**99.15**	**100.0**

ª The number of days absent from work due to category of comorbidity.

^b^Valued using GHS14.88 which is the national minimum wage as of January 2023.

^c^GHS11.63 equivalent to US$1.00 (BoG interbank exchange rate as of December 2023).

### Total economic cost of managing PLHIV and hypertension and diabetes

As shown in Table 8, the total treatment cost for HIV and comorbidities of hypertension and diabetes for the last six months was estimated at GHS11, 892.11 (US$1,022.54) with mean cost of GHS 212.36 (US$ 18.26). Out of the total treatment cost, hypertension only as a comorbidity was estimated at GHS 5,697.08 (US$ 489.86) with a mean cost of GHS 101.73 (US$ 8.75) while diabetes only as a comorbidity was estimated at GHS 2,663.58 (US$ 229.03) with a mean cost of GHS 47.56 (US$ 4.09). Of the total cost for hypertension only, direct cost accounted for GHS 5,017.00 (US$ 431.38). The direct cost of the total economic cost of managing PLHIV with comorbidities of hypertension and diabetes formed over 90% of the cost profile estimated at GHS10,739.00 (US$ 923.38) with a mean of GHS 191.77 (US$ 16.49). However, to estimate the annual total and average costs, each cost was multiplied by 2. Therefore, adjusting the six-month estimated total and mean direct cost associated with managing HIV, hypertension, and diabetes comorbidities to an annual basis. The estimated annual direct cost was GHS21,478 (US$1,846.76) with a mean of GHS383.54 (US$32.98). Also, the annual total and mean indirect cost amounted to GHS2,306.28 (US$198.30) and GHS41.18 (US$3.54) respectively. Furthermore, adjusting the total and mean cost associated with managing HIV, hypertension, and diabetes comorbidities annually amounted to GHS23,784.28 (US$2,045.08) and GHS424.72 (US$36.52) respectively. This includes the combined cost incurred across these health conditions.

**Table 8:** Total Cost of Managing PLHIV and Hypertension and Diabetes.

Cost category	Cost		Mean (GHS)	Cost profile (%)
	GHS	USD		
**Hypertension only:**				
Direct cost	5,017.00	431.38	89.59	42.2
Indirect cost	680.08	58.48	12.14	5.7
**Subtotal**	**5,697.08**	**489.86**	**101.73**	**47.9**
**Diabetes only:**				
Direct cost	2,421.00	208.17	43.23	20.4
Indirect cost	242.58	20.86	4.33	2.0
**Subtotal**	**2,663.58**	**229.03**	**47.56**	**22.4**
**Both Hypertension and diabetes:**				
Direct cost	3,301.00	283.83	58.95	27.8
Indirect cost	230.49	19.82	4.12	1.9
**Subtotal**	**3,531.49**	**303.65**	**63.06**	**29.7**
Direct cost	10,739.00	923.39	191.77	90.3
Indirect cost	1,153.14	99.15	20.59	9.7
**Total**	**11,892.15**	**1,022.54**	**212.36**	**100.00**

GHS11.63 equivalent to US$1.00 (BoG interbank exchange rate as of December 2023)

### Total economic cost as a percentage of GDP per capita


$$\frac{1022.54}{2260.3}x100=45.24\%$$

Using Ghana’s 2023 GDP per capita of US$ 2260.3 and the total economic cost of US$ 1022.54 for management of HIV and comorbidities of hypertension and diabetes over 6 months, the total economic cost as a percentage of GDP per capita was approximately 45.24%.

### Intangible cost of PLHIV with hypertension and diabetes comorbidities

**Fig 1:**
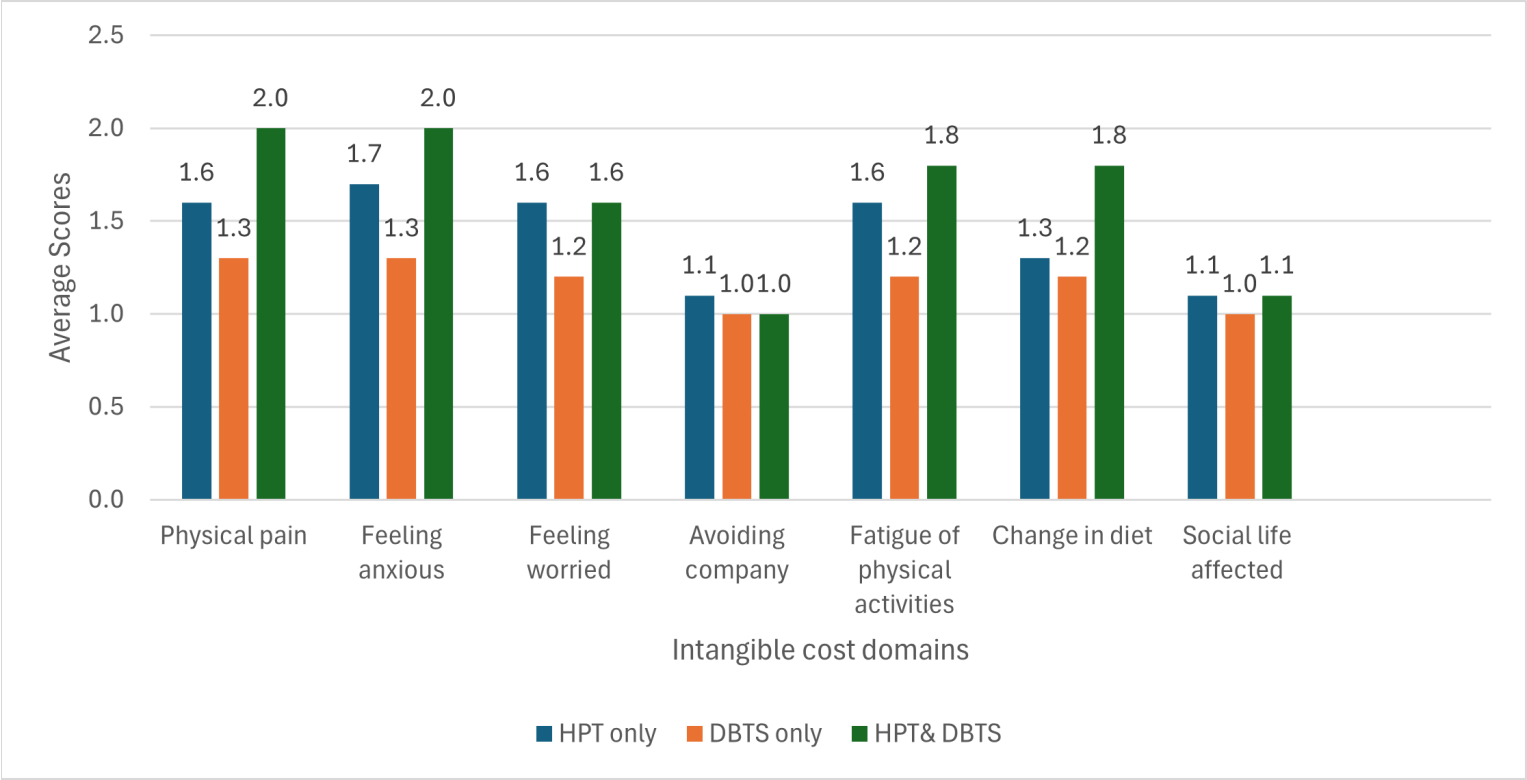
Average scores of intangible costs. HPT, hypertension; DBTS, diabetes.

As shown in Fig 1, participants with both hypertension and diabetes consistently reported higher intangible cost scores compared to hypertension only and diabetes only across all domains. Similarly, higher intangible costs were reported among participants with hypertension only compared to diabetes. However, for domains such as avoiding company and effect on social life, very marginal differences were observed across the comorbidities. The highest average intangible cost scores were in the domain of physical pain, feeling anxious, fatigue of physical activity and change in diet among participants who had both hypertension and diabetes.

### Composite intangible cost

**Fig 2:**
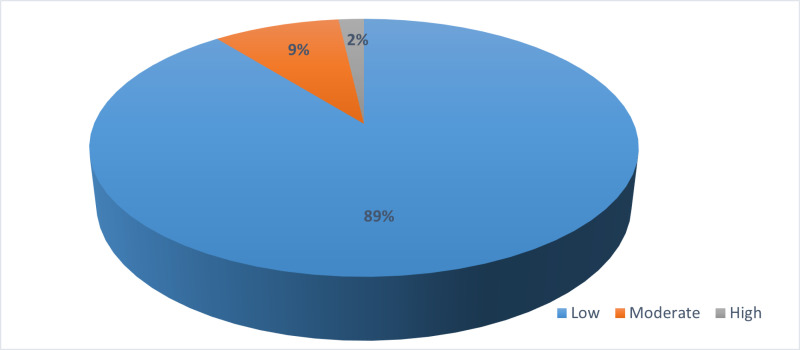
Composite intangible cost.

Fig 2 explains the overall intangible cost converted into three dimensions. These are high, moderate, and low. About 89% (n = 50) of respondents reported a low intangible cost burden, 9% (n = 5) respondents reported a moderate intangible cost burden, and about 2% (n = 1) reported a high intangible cost burden.

### Sensitivity analysis

The price of medicine was increased by 25% for all the conditions and this resulted in 18.8% and 15.2% increase in total cost of managing both hypertension and diabetes as comorbidities and hypertension only respectively. An increase in the daily wage to GHS44.54 which is the estimated mean daily wage of participants led to a 36.0% and 17.6% change in total cost burden for hypertension only as a comorbidity and diabetes only respectively. (Table 9)

**Table 9:** Sensitivity Analysis (One-way Sensitivity Analysis).

Item	Cost Component	Change in Parameter	Total cost		% change in total cost
			GHS	USD	
**Hypertension Only**					
Base scenario					
		–	5,697.08	489.86	–
Scenario 1	Medicine	25%	6,565.58	564.54	15.2
Scenario 2	Respondents mean daily wage	GHS 44.54	7,745.92	666.03	36.0
**Diabetes only**					
Base scenario		–	2,663.58	229.03	–
Scenario 1	Medicine	25%	3,088.58	265.57	16.0
Scenario 2	Respondents mean daily wage	GHS 44.54	3,133.64	269.44	17.6
**Both Hypertension and Diabetes**					
Base scenario		–	3,531.49	303.65	–
Scenario 1	Medicine	25%	4,193.99	360.62	18.8
Scenario 2	Respondents mean daily wage	GHS 44.54	4,199.59	361.10	18.9

## Discussion

The study found that about 57.1% of the patients had hypertension only as a comorbidity while 28.6% had diabetes only as a comorbidity and 14.3% had both hypertension and diabetes, thus hypertension was the commonest comorbidity seen among people living with HIV as compared to diabetes which is similar to findings by Sarfo et al. [31] in Ghana and Rajagopaul et al. [32] in South Africa. A global pooled prevalence of 25.2% of hypertension among PLHIV was reported in a systematic review involving 25 papers from America, 13 from Europe, 10 from Africa, and 1 from Asia [33]. A study in France on comorbidities of PLHIV also reported cardiovascular disease as one of the highest comorbidities (7.4%) in PLHIV. However, the proportion was lower as compared to the findings of this study [10]. The systematic review [33] had more papers from the Americas and Europe with few from Asia and Africa just as the study from France. These studies largely reflect high income economies thus may explain the lower proportions reported as compared to this study which was conducted in a Low-middle income country.

The majority of the patients (44.6%) were aged 50-59 years with the mean age of 53.20 years, which implies that hypertension and diabetes are more common among elderly HIV patients. This finding supports a study by Yang et al. [34] who suggested that comorbidities among people living with HIV are more pronounced in patients aged over 40 years. Females constituted about 76.8% of the study participants which could mean that more women were living with HIV than men. This prevalence of HIV among women has been attributed to the complex interplay of biological, social, and economic factors. This is in line with findings by Girum et al. [35] and Owusu’s [36] studies on gender disparities in HIV/AIDS. The Ghana AIDS Commission’s Fact sheet of 2019 shows an estimated 64% of females and 36% of males living with HIV [37]. Similarly, at the global levels, higher prevalence of hypertension was found in females than males [38] and the global age-standardized total diabetes prevalence was reportedly higher in females than in males as well according to the global burden of disease study [14].

The economic burden of managing PLHIV and comorbidities of hypertension and diabetes was substantial with the total economic cost of GHS11,892.14 (US$1,022.54). Total direct cost of GHS 10,739.00 (US$ 923.39) accounted for 90.3% of the total economic cost. A large part of the direct cost component was attributed to direct medical cost with a mean cost of GHS 159.27 (US$ 13.69) per patient and median of GHS 102.5 (IQR: 70-225). This finding aligns with previous studies that have identified medication as a major cost driver in the management of chronic conditions in Pakistan [39]. This study’s estimate of a high proportion (83.1%) of direct medical cost over total direct cost is attributed to the cost of medication for managing comorbidities in PLHIV. Thus, the cost of medication was estimated at GHS 3,474.00 (US$ 337.83), GHS 1,700.00 (US$ 146.17), and GHS 2,650.00 (US$ 227.86) for hypertension only, diabetes only and both hypertension and diabetes comorbidities respectively. Despite hypertension recording the highest total cost of medication, the median cost of medications for both hypertension and diabetes (GHS 155, IQR:150-470) was higher compared to hypertension only (GHS 80, IQR 50:-110) and diabetes only (GHS 87.5, IQR: 70-150). The higher total costs of medications for hypertension only as compared to diabetes only or hypertension and diabetes combined could be due to higher number of participants with hypertension only, the severity of conditions and type/number of medications each participant is placed on. Thus, the total costs could be influenced by the number of people incurring the expenses and type/number of medications.

On the other hand, higher median costs were reported for both hypertension and diabetes compared to hypertension and diabetes only. The comparison of total direct costs across comorbidities also revealed significant differences among the various conditions. It was seen that the costs of managing both hypertension and diabetes were significantly higher compared to having just one comorbidity. The higher median costs for managing both hypertension and diabetes reflect the compounded burden of double diseases. In general, this group is more likely to incur additional costs due to the multimorbidity. Thus, the median cost presents a better highlight of the financial burden experienced by patients. The result of this current study implies that PLHIV with both hypertension and diabetes comorbidity showed an increased cost burden compared to PLHIV with either diabetes or hypertension only as comorbidity. A systematic review in the United Kingdom reported similar results as seen in this study that, multimorbidity was associated with higher hospital, transition and total healthcare costs [40]. Contrary to the findings in this study and that in the United Kingdom, a study on costs of integrated HIV, diabetes and hypertension care in Tanzania and Uganda reported lower costs for combined management of hypertension and diabetes compared to managing one condition [41].

The cost of medications constituted about 32.3%, 15.83% and 24.7% of the total direct medical cost for hypertension only, diabetes only and both hypertension and diabetes respectively. Similar proportions were observed by Wierzejska et al. [42] who noted that in Jamaica, drug cost constituted 32% of the direct cost for general hypertension patients with any comorbidity. In the same study, it was found that Mexico and the USA reported drug costs of 14% and 15% respectively which is lesser than this study’s findings as the study was on general hypertensive patients. Given that the study’s focus was on general hypertension, patients without comorbidities could explain the lower medication cost reported as they did not incur expenditure on any comorbidity. In a parallel vein, a study undertaken by Butt at al. [39] disclosed that medication cost constituted 66.8% of the total direct cost for patients diagnosed with type 2 Diabetes Mellitus in Pakistan, albeit in the absence of HIV and hypertension. However, the findings of this current study contrast with study results by Poudel at al. [43] which reported that the cost of diagnostic tests for HIV patients in Nepal accounted for the highest proportion of direct cost. This discrepancy is because government-provided treatment in Nepal was limited to CD4 tests, while all other laboratory tests are paid out-of-pocket [43].

The total economic cost of managing HIV and comorbidities of hypertension and diabetes was about 45.24% of Ghana’s GDP per capita in 2023. The average cost of managing these comorbidities over six months translates to almost half the average annual income per person in Ghana using GDP per capita. This percentage highlights the vulnerability of affected households especially in low- and middle-income countries (LMICs). Schutte at al. [44] in a study reported that the proportion of mean daily household income per capita spent on maintaining healthy diets was 52% for lower-middle income, 89% for low-income, 28% for upper middle income and 6% for high come countries [44]. The expenditure figure for lower-middle income countries was similar to that found in this study. Although the study by Schutte et al. [44] was not a cost-of-illness study but rather a cost of consuming healthier diets, the findings could be closely compared due to their relationship with development of chronic NCDs. This expenditure could lead vulnerable populations into further poverty since that translates to more than 10% expenditure of income on healthcare [45].

Direct non-medical costs, although contributing to a smaller proportion of the total costs, were still significant. Direct non-medical costs accounted for 16.9% of the total direct cost at an estimated cost of GHS1,820.00 (US$156.49). Travel expenses to health facilities were the highest among non-medical costs, highlighting the geographic and financial barriers to accessing care. This finding aligns with previous research in low- and middle-income countries (LMICs), where transportation costs often present a significant barrier to accessing care, especially for patients living in rural or remote areas. For instance, a study by Eze et al. [46] in Nigeria found that transportation costs made up 25% of the total direct costs for patients managing chronic conditions. Also, in Uganda a study noted that transport costs were the largest contributor to indirect costs for all patients [47]. It highlights the importance of proximity to healthcare facilities and the potential benefits of decentralizing care to reduce travel-related barriers.

Patients incur significant medication expenses alongside the costs associated with traveling to and from healthcare facilities for treatment. The combined impact of these factors underscores the substantial financial burden borne by individuals managing these complex comorbidities. These findings emphasize the need for an integrated care model where patients with multiple conditions make one trip to the health facility for treatment rather than multiple visits. This has the potential to reduce frequency of hospital visits since patients can receive comprehensive care during a single visit, minimize travel costs and productivity losses and avoid the potential duplication of diagnostic tests. According to Duffy et al.[48], the integrated care could be non-communicable diseases (NCDs) care integrated into centers originally providing HIV care, or HIV services integrated into existing primary health care centers providing NCD services or simultaneous introduction of integrated HIV and NCD services [48,49]. In a scoping review in Africa, Chireshe et al. [49] reported that integrated care could offer short term benefits such as improved staff capacitation, improvement in patient retention, screening and detection of NCDs [49]. A study in Uganda and Tanzania noted that integration of HIV services with diabetes and hypertension substantially reduces health service and household costs [41].

The economic implications go beyond the direct costs of seeking care for the conditions. These implications encompass a wider range of indirect costs, particularly evident in the form of reduced productivity. This is demonstrated by instances of absenteeism and the compromised ability of patients to actively participate in productive work. The multidimensional nature of these indirect costs emphasizes the overall impact that the management of HIV and its associated conditions can have on individuals as well as the broader economic landscape.

The total indirect cost borne by patients and their accompanying caregivers to and from the hospital to seek care is estimated at GHS1,153.14 (US$99.15). This study’s finding revealed that people living with HIV and hypertension, HIV and diabetes, and HIV with both hypertension and diabetes lost an estimated 206 hours (33.2%), 65 hours (5.6%), and 47 hours (9.7%) respectively as productivity losses due to these conditions over six (6) months. The study’s finding is lower for combinations of PLHIV and diabetes and both hypertension and diabetes compared to findings from a study by Shiri et al. [41] in Tanzania and Uganda. This difference reported from Tanzania and Uganda might be attributed to their integrated health services for these conditions, in contrast to Ghana where the services for these conditions are provided separately. Mitchel and Bates [40] in their study have reiterated the value of maintaining a healthy population to prevent illness related productivity loss.

Additionally, out of the estimated 649 hours of lost productivity, 7.5% (47 hours), 2.5% (16 hours) and 1.6% (10 hours) were due to travel time in the last six months for a combination of HIV and hypertension, HIV and diabetes and HIV with both diabetes and hypertension. This translated to GHS86.96 (US$7.48), GHS29.14 (US$2.51) and GHS17.98 (US$1.55) respectively. This distribution of lost productivity hours highlights the impact of travel time on overall productivity loss among patients managing these comorbidities. Also, participants reported a low productivity loss due to waiting time as they visited the health facility based on scheduled appointments, resulting in reduced crowding and less waiting time.

The study participants reported high intangible cost scores for fatigue from physical activity, worry when taking medication, anxiety, and physical pain from HIV and hypertension and or diabetes comorbidities. This supports a study by Baye at al. [50] which confirmed that fatigue is a common occurrence among adults living with HIV in Ethiopia, but the study did not mention the comorbidities. This finding underscores the debilitating impact of chronic conditions such as HIV, hypertension, and diabetes on individuals’ energy levels and ability to participate in daily activities. Fatigue can impair physical functioning, diminish quality of life, and contribute to feelings of frustration. Multiple conditions demand multiple medication intake, hence patients managing multiple chronic conditions may grapple with concerns related to treatment effectiveness, the long-term management of their health conditions and uncertainty about their future health outcomes.

Furthermore, anxiety is a common mental health challenge faced by individuals managing chronic illnesses, and it can manifest as persistent feelings of apprehension, fear, or unease [51]. Anxiety can negatively impact overall well-being, exacerbate physical symptoms, and interfere with daily functioning and coping mechanisms. Chronic pain can significantly diminish quality of life, impair mobility, and exacerbate psychological distress [52]. Managing physical pain requires a multifaceted approach that addresses both the underlying health conditions and the individual’s pain management strategies. The result also suggests that, on average, patients have fewer intangible costs associated with modifying their dietary habits to manage their health, maintaining their social connections and engagements, and isolating themselves from others because of their illnesses.

### Limitations of the study

The study provides valuable insights into the costs of managing hypertension and/or diabetes among PLHIV. However, some limitations exist. First, the relatively small number of participants may limit the generalizability of the findings to the broader population of people living with HIV and comorbidities of hypertension and diabetes. Second, the reliance on self-reported data for the past 6 months could introduce recall bias which could affect the accuracy of the cost estimates. Participants may have underreported or over reported their expenses. Due to the paucity of published literature on costs of HIV and comorbidities, the limited ability to make broader range of comparisons underscores a significant gap in the current body of research. Despite these limitations, this study contributes to knowledge as it remains one of the few studies that have estimated the economic burden of managing PLHIV and comorbidities of hypertension and diabetes in Africa. More importantly, to the best of the author’s knowledge, this is the first study in Ghana, to assess the economic burden of this population in the Ghanaian context.

## Conclusion

The study highlights the substantial economic burden placed on households in managing HIV and comorbidities of hypertension and diabetes. Direct medical costs, which were mainly driven by costs of medications and indirect costs largely due to productivity loss by both patients and caregivers, further compounded the financial burden on households particularly for those managing both hypertension and diabetes. The total economic cost constituted about 45.24% of Ghana’s GDP per capita, highlighting the substantial financial burden on affected households.

Although health insurance subscription was high, significant out of pocket expenditures are made emphasizing the need for targeted and more comprehensive policies such as holistic coverage of care under the National Health Insurance Scheme to support and reduce financial barriers to the management of chronic diseases. The findings underscore the critical need for integrated, cost-effective strategies that address both communicable and non-communicable diseases in low- and middle-income settings. This may include access to integrated healthcare services, holistic management of comorbidities, programmes aimed at improving the quality of life of PLHIV with more focus on the elderly, increasing access to affordable medications, improving adherence to treatment, and minimizing productivity losses through more flexible healthcare delivery systems. Future research should explore the long-term economic burden of managing communicable diseases with non-communicable comorbidities, associated coping strategies and assess effectiveness of strategies aimed at reducing the economic burden among vulnerable populations.
